# Modern Sociology

**Published:** 1902-09-13

**Authors:** 


### Modern Sociology.

### "SWEATING NUNS."

A RATHER curious case lately came before the Court of Cassation in Paris. The nuns of the Convent of the Good Shepherd, at Nancy, do a great deal of both plain and fancy sewing. The orders they receive are so numerous that they cannot execute them by themselves, and engage lay women and girls to assist. They are charged with overworking these lay helpers and paying them hardly anything. We have heard of similar complaints, though we cannot say whether they are justified or not, being made regarding the convents in Ireland where lace is made, not only by the sisters but by the children whom they are educating. Convents are very largely managed on business principles now. Several of those in Normandy and Brittany do a large boarding-house business during the summer months and make no trouble over either the sex or religion of their guests. Almost all have some speciality in work, be it embroidery, lace, or confectionery making, and the convent orphanages are supported not only by the gifts of the faithful, but by the payments of parishes which board out their pauper children of the same creed under the care of the nuns. One would naturally assume that women who devote their lives to the service of God as sincerely and fervently as nuns of all kinds do, would conduct their establishments on the best possible principles, but, unfortunately, this same religious fervour tends to make them lose sight of the needs and capacities of the instruments employed. If the mother superior and the cloistered sisters under her are willing to work night and day for the benefit of their Church, they are apt to expect a like devotion from their unvowed helpers, forgetting that these want only to make a living, and will not count it gain if life is shortened through overwork, as a nun might do. A religious enthusiast, without having any intention of cruelty, is often hopelessly inconsiderate towards others. Reports of overwork having gone about regarding the Nancy Convent, an inspector went there at nine o'clock one night to see if there was any foundation for them. The superior of the convent, however, refused to admit him, and for this she was fined 100 francs by the Nancy tribunal. She appealed against the decision, and obtained one in her favour from a higher court, which declared that there was no sufficient reason for the night visit of the inspector. Against this decision, in turn, the Procureur-General at Nancy appealed, but at the Court of Cassation the verdict was again in favour of the nuns. While, so far, victory has gone with the convent, the case may very probably raise a question in the public mind, which will want to know why, if convents, or other philanthropic or religious establishments, undertake outside work, they should not be regarded simply as factories, and placed under the ordinary limitations of the Factory Acts, obtaining no advantages in this respect from the fact that they are religious foundations?

### TRAINING IN OBSERVATION.

THE School Boards, or whatever authority takes the place of School Boards in South Germany, have ideas on education beyond what these bodies have here. Not content with instructing the children under their charge, they try to find out what native capacity their pupils have. Lately they made an experiment with a view to testing their power of observation. A man in the clothes of an ordinary workman was placed in a room, and the boys and girls of the school were marched through it. Afterwards they were examined as to what they remembered of the man who had been in the room. And this inquiry revealed a notable sex-quality. Of the girls, nearly 80 per cent. confined their comment to the man's clothes, while only the remaining 20 per cent. were able to tell something of his features in addition. With the boys the reverse was the case. Seventy per cent. of them could describe the man's features, but had paid no attention to his dress, while the remainder knew something of both. Apart from the curious light which this experiment throws on what children see when they look, and the line of education which it points out as desirable to be pursued in the case of boys and girls respectively, the knowledge of what children may be expected to observe has a rather important bearing on the credibility of young persons as witnesses in a court of law. It is clear that if these children were pretty much like their neighbours in the point of observation, girls are not to be relied on to recognise a person whose dress has been changed. The features of a person they meet do not catch their eye, unless, indeed, they be specially remarkable. But to a boy the face is the important matter, and the clothes matter little. Therefore, it must be inferred that where a question of individuality arises their testimony is more to be relied on than that of their girl companions.

### VAGABONDIA.

"THE world is Vagabondia to him who is a vagabond," sings Bliss Carman, and though it may seem hard for even the most determined vagabond to carry out his aims in a civilised and thickly populated country like ours, it is not impossible. We do not mean the tramp who seeks the shelter of the casual ward at the end of his journeying, but the man who, day in and day out, prefers rather to "hear the lark sing than the mouse squeak." Such we may reckon the old sailor who made his home in an old upturned boat—not made comfortable, like Peggotty's, but in the rough, save for some old bits of tarpaulin, and located below high-water mark. The owner of the ground above high-water mark had objected to the presence of this maritime squatter, and obtained an order for his removal, but by shifting his home a few feet nearer the edge of the sea, the old salt was able to defy all interference of private individuals. Even more defiant was he who took up his abode, with his family, in the holes on a common. No one had the right to dislodge him, for he was on common ground. The parish council of the neighbourhood indeed tried to proceed against him for being without visible means of subsistence; but when an attempt was made to eject him on that score he pulled some sovereigns out of his pocket, and the attempt was thus proved futile. Very annoying to the feelings of sensitive people are such folks, yet not altogether without a possible usefulness as citizens. Recently a journalist met an invalided soldier whose face seemed to be familiar to him. On inquiry he found that the soldier was one with whom he had made acquaintance on a walking tour in a previous summer, and who followed the trade of a tinker. The ex-tinker told his questioner that a good many of his trade had joined the Army and liked the life at the front very well. Moreover, the sleeping on the bare veldt and other things that seemed hardships to those brought up in more luxurious surroundings, were no hardships to the tinkers. It was a wound, not sickness, that had sent him home, and he was willing to go out again as soon as he was fit. Nothing, so far as is recorded, was said of the tinker's superiority in foraging; but perhaps in this also he surpasses his fellows. At any rate, the tinker seems to have found a place for himself in South Africa.

